# Assessing the Impact of Nutritional Stress on the Identification of Plastic-Associated Bacteria in Insect Gut Microbiota

**DOI:** 10.3390/microorganisms14030649

**Published:** 2026-03-13

**Authors:** Kenza Dessauvages, Grégoire Noël, Alexandre Verdin, Joachim Carpentier, Frank Delvigne, Gauthier Eppe, Frédéric Francis

**Affiliations:** 1 Functional and Evolutionary Entomology, Gembloux Agro-Bio Tech, University of Liege, Passage des Déportés 2, 5030 Gembloux, Belgium; kenza.dessauvages@uliege.be (K.D.); gregoire.noel@uliege.be (G.N.); joachim.carpentier@uliege.be (J.C.); 2 Mass Spectrometry Laboratory, University of Liège, 4000 Liège, Belgium; alexandre.verdin@uliege.be (A.V.); g.eppe@uliege.be (G.E.); 3 Microbial Processes and Interactions, Gembloux Agro-Bio Tech, University of Liege, Passage des Déportés 2, 5030 Gembloux, Belgium; f.delvigne@uliege.be

**Keywords:** gut microbiota, nutritional stress, plastivorous insects, symbiosis, bacterial communities

## Abstract

The plastic-degrading capacity of some insects has been investigated over the past decade, with the aim of identifying gut microorganisms potentially involved in plastic degradation. However, plastic-only diets impose severe nutritional constraints, potentially driving microbial selection independently of plastic exposure. Here, we examined how nutritional stress influences gut bacterial community and the identification of plastic-associated bacteria in two plastivorous insects, *Galleria mellonella* and *Tenebrio molitor*, using polyurethane (PU) as a representative polymer. Bacterial communities were characterized by 16S rRNA gene sequencing under contrasted dietary conditions, including starvation, and complemented by a culture-dependent isolation approach using PU as the sole carbon source. In both species, gut bacterial communities under plastic-only feeding closely resembled those observed under starvation, whereas they differed from nutritionally balanced conditions. Differential abundance analyses reflected this pattern, as taxa enriched under plastic feeding were also enriched under starvation. This convergence was strong and structured in *T. molitor*, but weaker and more variable in *G. mellonella*. In addition, bacterial strains were isolated from the gut of *T. molitor* under both PU-amended and carbon-free conditions. Overall, our results demonstrate that nutritional stress is a driver of gut bacterial community restructuring under plastic-based diets and can bias the identification of candidate plastic-associated bacteria.

## 1. Introduction

Global plastic production and consumption have increased steadily over the past decades. Many materials have been replaced by plastic polymers in a variety of sectors. Their versatility, low cost, light weight, and strength make them indispensable materials in our modern lifestyle [1]. Every aspect of our daily lives is affected: transportation, construction, electronic devices, food packaging, and even healthcare—plastics are now ubiquitous. However, these advantageous properties translate into significant challenges at the end of their life cycle [2].

Current methods of plastic waste management have significant limitations: incineration and landfilling cause high levels of pollution, recycling remains costly and inefficient, and a significant proportion of waste still escapes treatment channels and ends up in the environment. The consequences of this accumulation are now well documented and constitute a major environmental problem [3,4].

Faced with this situation, exploring alternative biological approaches, particularly those based on the action of enzymes, is emerging as a promising and more sustainable strategy. These biocatalysts are capable of breaking down plastic polymers into simpler compounds, paving the way for bio-upcycling, which is the transformation of plastic waste into value-added chemicals [5,6]. Several organisms have demonstrated their ability to degrade plastics. Among them, some insects have attracted particular interest due to their ability to ingest and degrade polymers, a process that appears to involve their gut microbiota [7,8].

The gut microbiota of insects plays a central role in their physiology, behavior, and ecological adaptation. These symbiotic microbial communities participate in the digestion of hard-to-break-down substances, compensate for nutrient-poor diets, modulate the immune system, and protect their hosts against predators, parasites, and pathogens [9]. They also influence key functions such as dietary specialization, reproduction, and intra- and interspecific communication, thereby contributing significantly to the evolutionary and ecological success of insects. In both natural and anthropized ecosystems, these microbiomes also play an essential role in the decomposition of organic matter and biogeochemical cycles, particularly the nitrogen cycle [10,11].

Beyond their traditional physiological functions, insect gut microbiota is attracting increasing attention for their potential in degrading persistent pollutants, such as pesticides and plastics. Certain insect species such as *Tenebrio molitor* L., 1758 and *Galleria mellonella* (L., 1758) are widely used as model organisms because of their ability to ingest and alter different types of polymers, a phenomenon likely linked to the enzymatic activity of their intestinal flora [12]. Identifying the microorganisms involved is a critical step toward understanding the microbial mechanisms underlying plastic degradation in insects. To this end, several approaches have been employed, including the analysis of microbiota shifts associated with plastic consumption using 16S rRNA gene sequencing, as well as culture-dependent methods based on the isolation of microorganisms on media with plastic as the sole carbon source.

Despite the growing number of studies employing these approaches [2,13], disentangling the direct effects of polymer consumption from confounding dietary factors remains challenging as plastics have a low nutritional value, as evidenced by weight loss in insects fed exclusively on plastic [14]. This alone can drive major changes in microbiota composition and select for stress-tolerant or opportunistic microorganisms. As a result, it is often unclear whether the observed microbial changes are specifically induced by polymer degradation or simply reflect general microbial responses to nutritional stress. This methodological ambiguity undermines the robustness of candidate polymer-degrading microorganism identification and increases the risk of false-positive associations.

In this study, we investigated gut microbiota responses, focusing on bacterial communities, in two widely used plastivorous insect models, *G. mellonella* and *T. molitor*, using polyurethane (PU) as a case study. PU remains comparatively understudied relative to other commonly investigated plastics such as polyethylene or polystyrene, despite its widespread industrial use and environmental concerns [15]. To evaluate the influence of nutritional context on microbiota-based inferences, we incorporated appropriate nutritional controls in both gut microbiota profiling and culture-based isolation experiments. This approach allowed us to assess the extent to which nutritional stress alone shapes microbial community structure and biases the identification of bacterial taxa associated with plastic-based substrates.

## 2. Materials and Methods

### 2.1. Plastic and Insect Rearings

The polymer used in this study was a commercially available polyisocyanurate foam from the IKO Enertherm product line, belonging to the polyurethane (PU) family, purchased at Roof Isolation (Gembloux, Belgium). As the material is a commercial product, its exact chemical formulation is not publicly disclosed. The polymer was characterized by Attenuated Total Reflectance-Fourier Transform Infrared (ATR-FTIR) spectroscopy using a Nicolet iS5 spectrometer (Thermo Fisher Scientific, Waltham, MA, USA) equipped with an iD7 ATR accessory with a germanium (Ge) crystal. The corresponding spectrum is provided in the Supplementary Material (Figure S1).

*G. mellonella* and *T. molitor* larvae used in this study originated from laboratory colonies maintained at the Functional and Evolutionary Entomology laboratory of Gembloux Agro-Bio Tech (University of Liège, Belgium). They were co-reared in a controlled environmental chamber maintained at 29 °C ± 2 °C under complete darkness to simulate their natural habitat. The two species were maintained on their respective standard laboratory diets, reflecting their distinct ecological feeding habits, in order to ensure optimal development. *G. mellonella* larvae were fed an artificial diet, adapted from a previously described protocol [16], consisting of whole wheat flour, honey, powdered milk, polenta, brewer’s yeast, and glycerol, blended in precise proportions. *T. molitor* larvae received a diet composed of a 1:1 (*v*/*v*) mixture of wheat bran and whole meal flour, two substrates commonly used for mealworm production [17]. In parallel, we established an additional *T. molitor* colony in which larvae were supplemented with PU blocks over several generations.

### 2.2. Plastic Feeding Experiments and Sample Preparation

To investigate changes in the bacterial gut microbiome of larvae exposed to PU, both *G. mellonella* and *T. molitor* larvae from the standard rearings were selected at the late larval stage based on size (approximately 2 cm in length). Selected larvae originated from the same cohort, such that individuals of similar size were also of comparable age and developmental stage. These larvae were then subjected to four feeding treatments established based on preliminary tests and applied consistently to both species to allow for direct comparison. Groups of 25 larvae were placed in individual containers (18 × 18 × 7.5 cm). The treatments included a nutritionally complete control diet similar to the rearing diets (different for both insects; hereafter referred to as Control), a diet consisting solely of PU provided as small blocks (PU), a mixed diet composed of equal amounts of control diet and PU blocks (Mixed), and a starvation condition in which no food was provided (Starved). Larvae were maintained under these conditions for seven days, a duration limited by the life cycle of *G. mellonella*, which pupates shortly after, but sufficient to allow comparative analysis with *T. molitor*, which could tolerate longer exposures. This duration and the selected larval size were determined through preliminary tests under our rearing conditions to ensure sufficient time before pupation while maximizing larval size and feeding activity. Only individuals that remained at the larval stage and showed no visible signs of pre-pupation were selected for gut bacterial community analysis. After the exposure period, six larvae of each treatment were randomly selected, placed in 50 mL Falcon tubes, rapidly frozen in liquid nitrogen, and stored at −80 °C until further processing for gut microbiome analyses. The remaining larvae from each treatment were retained for plastic ingestion verification (in Section 2.3). Prior to gut dissection, larvae were surface sterilized by immersion in 70% ethanol for 30 s, followed by three rinses in sterile distilled water of 30 s each. Dissections were then performed under sterile phosphate-buffered saline (PBS). For each dietary treatment, three independent containers were prepared. From each container, two gut subsamples were collected. Each subsample consisted of pooled guts from three larvae originating from the same container. The two subsamples were processed and sequenced separately. The entire experimental setup was then independently repeated once for each insect species.

### 2.3. Plastic Ingestion Verification

At the end of the feeding exposure, plastic ingestion was assessed using the larvae that were not frozen for microbiome analyses. For each dietary condition, the remaining larvae were placed in a clean container, free of any debris, and their frass was collected after 24 h. The excreta were then treated with a 10 wt% potassium hydroxide (KOH) solution for one week at room temperature under continuous magnetic stirring to ensure homogeneous digestion of organic material while preserving potential PU fragments. After digestion, the residual solution was directly vacuum-filtered through an alumina filter with porosity of 0.2 µm. The remaining particles were observed under an optical microscope coupled with a Raman spectrometer. Raman spectra were acquired using a LabRAM 300 spectrometer (Horiba Jobin Yvon, Villeneuve-d’Ascq, France) interfaced with an Olympus BX40 confocal microscope (Olympus, Tokyo, Japan). Spectra were recorded under 532 nm laser excitation with two accumulations of 30 s using a 50× objective (NA 0.5) and a 1800 grooves/mm grating. Spectra were baseline-corrected using a fourth-degree polynomial fit. The recorded spectra and optical microscope images were compared with reference PU to confirm polymer identity.

### 2.4. Gut Bacterial Community Analysis: DNA Extraction and 16S rRNA Gene Amplification

Total DNA was extracted from pooled larval gut samples using the PowerFecal Pro DNA extraction kit (Qiagen, Hilden, Germany) following the manufacturer’s instructions with the following modifications: an additional incubation step at 65 °C for 10 min was introduced after the addition of the lysis buffer, and D-type beads were added alongside the supplied beads for enhanced sample homogenization during bead beating (MM400, Retsch, Haan, Germany). The total DNA concentration was quantified using a NanoDrop spectrophotometer (Thermo Scientific, Waltham, MA, USA).

PCR amplification was performed using species-specific DNA input conditions optimized to ensure reliable amplification using a C1000 Touch Thermal Cycler (Bio-Rad Laboratories, Hercules, CA, USA). The V3–V4 hypervariable region of the 16S rRNA gene was amplified by PCR using the universal primers 341F and 805R, with Illumina overhang adapters. PCR amplification was performed with KAPA HiFi HotStart DNA Polymerase under the following conditions: initial denaturation at 95 °C for 3 min, followed by 35 cycles of 98 °C for 20 s, 57 °C for 15 s, and 72 °C for 30 s, with a final extension step at 72 °C for 1 min. PCR products were purified using the NucleoSpin Gel and PCR Clean-up kit (Macherey-Nagel, Düren, Germany). Sequencing was performed on an Illumina MiSeq platform (Cornell University, Ithaca, NY, USA) using the MiSeq v2 500-cycle kit, generating paired-end reads of 2 × 250 bp.

### 2.5. Enrichment Culture and Bacteria Isolation and Identification from T. molitor Gut

To isolate bacteria potentially involved in PU degradation from the gut of *T. molitor*, we used larvae that had been reared with PU supplementation for three consecutive generations. After a 24 h fasting period, eight larvae were surface sterilized and their digestive tracts were aseptically dissected, as previously described, and pooled into a 1.5 mL Eppendorf tube containing 1 mL of sterile phosphate-buffered saline (PBS) using sterile forceps. The tissues were then homogenized with a polypropylene pestle compatible with 1.5 mL tubes until a uniform suspension was obtained. This homogenization step enhances the release of bacteria attached to the mucosa for further culture. The resulting suspension was centrifuged at 4000× *g* for 30 s using a benchtop microcentrifuge (Sigma Laborzentrifugen, Osterode am Harz, Germany) to pellet tissue debris while leaving the majority of bacteria in the supernatant.

One hundred microliters of this supernatant were inoculated into 100 mL of minimal salt medium (MSM) in a 200 mL Erlenmeyer flask. The MSM was composed of 0.7 g/L KH_2_PO_4_, 0.7 g/L K_2_HPO_4_, 0.7 g/L MgSO_4_·7H_2_O, 1.0 g/L NH_4_NO_3_, 0.005 g/L NaCl, 0.002 g/L FeSO_4_·7H_2_O, 0.002 g/L ZnSO_4_·7H_2_O, and 0.001 g/L MnSO_4_·H_2_O. The culture was supplemented with a piece of PU foam of approximately 5 × 1 × 0.3 cm as the sole carbon source. The PU had been previously sterilized by immersion in 70% ethanol for 1 h, rinsed several times with sterile distilled water, and then air-dried under a laminar flow hood. Two control cultures were also included: one consisting of 100 mL of MSM and a sterile piece of PU foam (without inoculum) to verify the effectiveness of the PU sterilization process, and another containing 100 mL of MSM and 100 µL of *T. molitor* digestive tract inoculum without PU to assess the potential growth of microorganisms in the absence of an external carbon source. All flasks were incubated under shaking conditions (30 °C, 100 rpm) to ensure proper aeration and contact between the microorganisms and the polymer substrate for 45 days using a thermostatic shaking incubator (C. Gerhardt GmbH & Co. KG, Königswinter, Germany). To prevent medium loss due to evaporation while maintaining aerobic conditions, a piece of paraffin was placed at the opening of each Erlenmeyer flask.

After 45 days of incubation, 100 µL of the medium was spread onto LB agar plates and incubated at 30 °C to allow colony development, with three replicates performed for each condition. Distinct colonies were successively subcultured on fresh LB agar plates until pure bacterial cultures were obtained. Each pure isolate was then grown in liquid LB medium for 24 h at 30 °C with agitation (100 rpm). Cultures were subsequently centrifuged at 4000× *g*, and genomic DNA was extracted from the resulting pellets using the PowerFecal Pro DNA Isolation Kit (Qiagen, Hilden, Germany). The V3–V4 hypervariable region of the 16S rRNA gene was amplified by PCR using the same primer pair employed for gut bacterial community analysis. PCR amplification was performed with KAPA HiFi HotStart DNA Polymerase under the following conditions: initial denaturation at 95 °C for 3 min, followed by 35 cycles of 98 °C for 20 s, 67.9 °C for 15 s, and 72 °C for 30 s, with a final extension step at 72 °C for 2 min. PCR products were purified using the NucleoSpin Gel and PCR Clean-up kit. Sanger sequencing was then performed by Eurofins Genomics (Cologne, Germany). Sequence chromatograms were checked and edited using Serial Cloner, and the resulting consensus sequences were compared to the NCBI GenBank database using BLASTn for taxonomic identification. Sequences showing ≥ 98% identity with reference sequences were considered to represent the same bacterial species.

### 2.6. Statistical Analysis

Demultiplexed paired-end sequencing reads were processed using QIIME 2 version 2024.10 [18]. Initial quality control involved trimming low-quality bases and filtering reads based on quality scores. Paired-end reads were then merged, and chimeric sequences were identified and removed using the DADA2 plugin, which also denoised the data and generated high-resolution amplicon sequence variants (ASVs). Taxonomic classification of ASVs was performed using a naïve Bayes classifier trained on the SILVA 138 database [19], specifically targeting the V3–V4 region of the 16S rRNA gene. Resulting ASV tables were imported into R for downstream analyses [20]. Subsamples originating from the same experimental container were then aggregated to match the experimental unit, by summing ASV counts of the two subsamples per container. All subsequent analyses were therefore performed at the container level.

Bacterial community analysis was performed using the phyloseq R package integrating the ASVs community matrix, ASVs taxonomy, the phylogenetic tree and the associated metadata [21]. The phyloseq object was then converted to a MicrobiotaProcess object for further analysis [22]. Rarefaction curves and the alpha diversity metrics (i.e., bacterial taxa richness or ASVs richness, Chao1’s estimator and Shannon index) were estimated by a split number of 100 chunks using the mp_cal_rarecurve function [22].

Beta diversity was evaluated using Bray–Curtis dissimilarity and visualized with principal coordinates analysis (PCoA) to examine differences in microbial community composition between treatments. Statistical significance of differences in beta diversity among groups was tested using permutational multivariate analysis of variance (PERMANOVA) with 999 permutations. We performed pairwise differential abundance testing of microbial taxa across four diet groups using a DESeq2 R package [23]. Prior to modeling, ASVs features were aggregated at the Genus level (when taxonomy was available) and filtered to retain taxa with ≥10 total reads and presence in ≥10% of samples. For each insect, count data were modeled with a negative binomial generalized linear model comparing our four experimental diet. Size factors were estimated with the poscounts method to accommodate zero-inflated microbiome counts, and models were fit with the parametric dispersion trend in DESeq2. All pairwise contrasts among the four diet levels were evaluated by releveling and extracting the appropriate coefficient per comparison. Log2 fold-changes (log2FC) were stabilized via shrinkage using the apeglm R package [24], and statistical significance was determined by controlling the false discovery rate at α = 0.05 (Benjamini–Hochberg adjusted *p*-values). For interpretability, we computed group-wise mean normalized abundances for the contrasted diets and joined available taxonomic annotations to each ASVs feature. Results were summarized as a heatmap of log2 fold-change values across dietary contrasts, with data handling performed using the tidyverse package and heatmap visualization generated with pheatmap.

## 3. Results

### 3.1. PU Ingestion Verification

Raman spectroscopy was used to verify the ingestion of polyurethane (PU) by both insect species. PU-derived particles were detected in frass from insects exposed to PU-containing diets, whereas no such particles were observed in samples from control or starved treatments. Raman spectra obtained from these particles displayed characteristic bands consistent with those of the reference PU material (Figure S2), confirming their polymeric identity. Given the nature of the commercial PU foam and the sensitivity of the measurement, Raman analysis was used here to verify material identity and does not allow detection of subtle modifications in particle composition.

Optical microscopy observations further revealed that the recovered particles retained the typical alveolar structure of the pristine PU foam (Figure S3), indicating that the material recovered from frass corresponded to fragmented PU.

These results demonstrate that PU is ingested and transits through the digestive tract of both *G. mellonella* and *T. molitor* under PU-feeding conditions.

### 3.2. Baseline Differences in Gut Bacterial Community Diversity Between Species

Illumina MiSeq sequencing of the V3–V4 region of the 16S rRNA gene generated a total of 9,542,384 paired-end reads across all samples, with a mean of 107,218 reads per sample.

**Figure 1:**
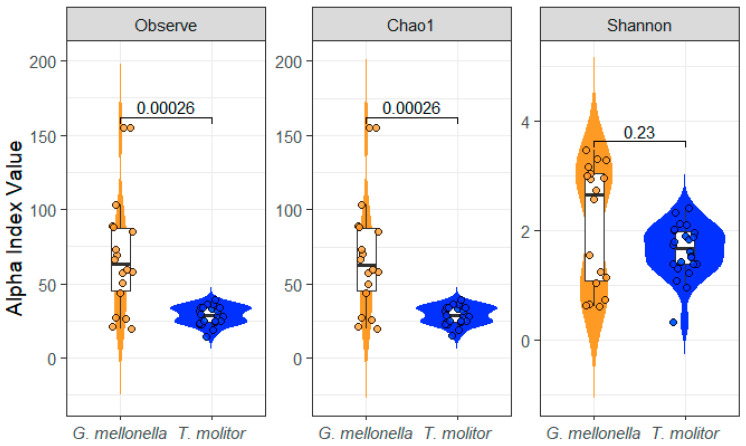
Alpha-diversity indices of gut bacterial communities in *G. mellonella* (orange) and *T. molitor* (blue). Observed richness, Chao1, and Shannon index values were calculated across all samples from both species. *p*-values are reported for each species comparison.

An initial comparison of gut bacterial community diversity between the two insect species showed clear differences between *G. mellonella* and *T. molitor*. 299 taxa were specific to *G. mellonella* and 40 to *T. molitor*, whereas 81 taxa were shared between the two species. Alpha diversity of gut bacterial communities differed significantly between *G. mellonella* and *T. molitor* for richness-based indices (Figure 1). Observed richness was significantly higher in *G. mellonella* than in *T. molitor* (*p* = 0.00026). A similar pattern was found using the Chao1 estimator, confirming a greater estimated taxonomic richness in *G. mellonella* (*p* = 0.00026). In contrast, the Shannon diversity index, which accounts for both richness and evenness, did not differ significantly between the two species (*p* = 0.23), although *G. mellonella* showed higher and more variable values.

These results indicate that *G. mellonella* harbors a richer bacterial community within its gut microbiota in terms of taxon number than *T. molitor*, while the overall distribution of abundances among taxa remains broadly similar between the two species.

### 3.3. Dietary Impact on Gut Bacterial Community Diversity and Structure

**Figure 2:**
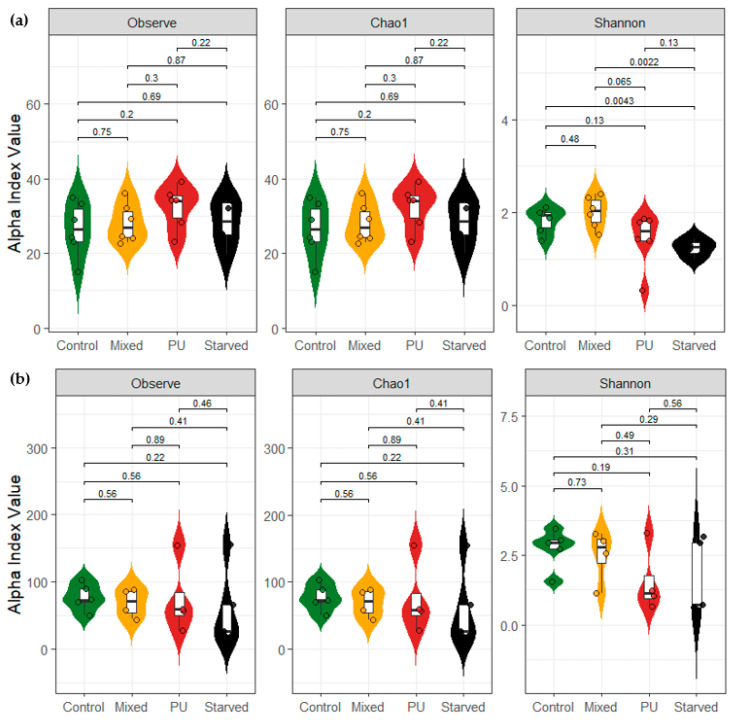
Alpha diversity indices across four diet treatments in (**a**) *T. molitor* and (**b**) *G. mellonella*. Observed richness, Chao1, and Shannon index values were calculated across all samples per species and diet. *p*-values are reported for each pairwise comparison.

Alpha-diversity metrics remained largely similar across diets, with only limited differences in *T. molitor*. For both species, Observed richness and Chao1 showed very similar distributions among the four dietary treatments, with all reported *p*-values being non-significant (Figure 2). For *T. molitor*, Shannon diversity is lower in the Starved group, with significant differences between the Mixed and Starved groups, as well as between the Control and Starved groups. Shannon values also tend to be lower in the PU group compared to the Mixed diet, although this difference does not reach statistical significance (*p* = 0.065) (Figure 2a). For *G. mellonella*, Shannon diversity values overlap across Control, Mixed, PU, and Starved groups, and all pairwise comparisons yield non-significant *p*-values (Figure 2b). Overall, diet effects on alpha diversity are minimal and primarily driven by starvation in *T. molitor*, whereas *G. mellonella* shows no detectable alpha-diversity response across diets.

**Figure 3:**
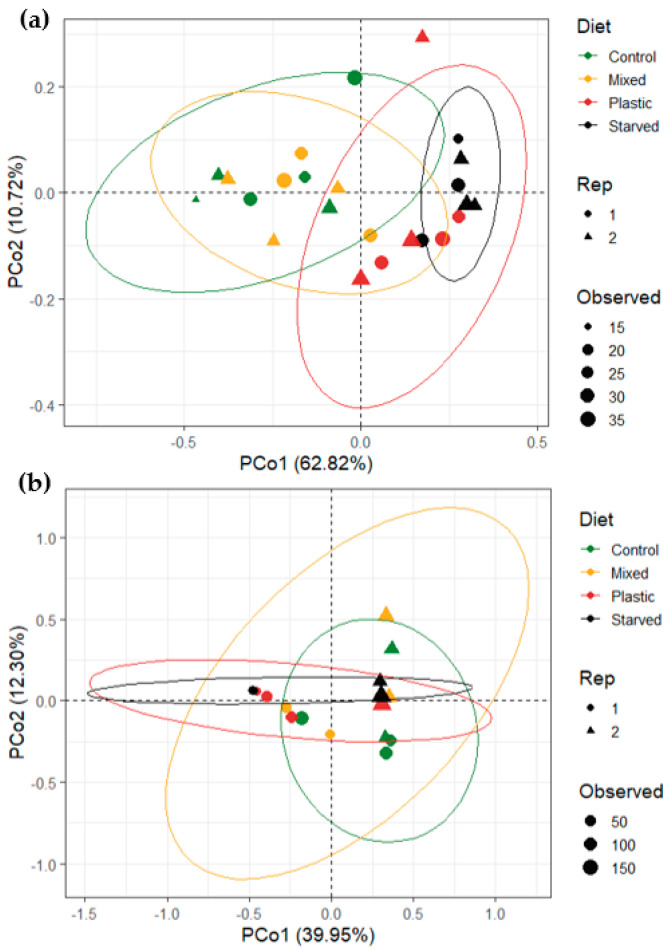
Principal coordinates analysis (PCoA) of gut bacterial community composition in (**a**) *T. molitor* and (**b**) *G. mellonella* under the four different diet treatments: the control diet, the mixed diet, the PU diet and starved group. Ordinations are based on Bray–Curtis distances calculated from Hellinger-transformed ASV relative abundances. Points represent individual samples, colored by diet and shaped according to experimental repetition (Rep 1 and Rep 2). Point size is proportional to observed richness. Ellipses represent 95% confidence intervals around dietary group centroids.

The PCoA shows a clear structuring of *T. molitor* gut bacterial communities according to diet (Figure 3a). The first two axes explain 62.82% (PCo1) and 10.72% (PCo2) of the total variation, respectively. Samples from the Control and Mixed diets largely overlap and cluster on the negative side of PCo1, indicating similar community composition between these two treatments. In contrast, samples from the PU and Starved groups are shifted toward positive values of PCo1, forming clusters that are separated from Control and Mixed samples. The Starved group shows a relatively tight clustering, whereas the PU group is more dispersed, indicating higher inter-individual variability under the plastic diet. Some overlap remains between PU and Starved samples, but both groups are clearly separated from Control and Mixed along the main ordination axis. PERMANOVA confirms a strong effect of diet on community composition (Diet: R^2^ = 0.532, F = 7.63, *p* = 0.0001), while experimental repetition has no significant effect (Rep: R^2^ = 0.027, F = 1.16, *p* = 0.28).

In *G. mellonella*, PCoA did not reveal a clear structuring of gut bacterial communities according to diet, as samples from the different dietary treatments largely overlapped (Figure 3b). Despite this visual overlap, PERMANOVA detected a significant effect of diet on community composition (Diet: R^2^ = 0.227, F = 1.93, *p* = 0.0281), indicating that diet explained a measurable proportion of the variance. In contrast, on the PCoA plots, samples showed a clearer separation according to experimental repetition (Rep). This is confirmed by PERMANOVA, which identifies repetition as a major explanatory factor (Rep: R^2^ = 0.242, F = 6.17, *p* = 0.0007), explaining more variance than diet. On the PCoA plots, samples from the first repetition show a partial separation by diet, whereas samples from the second repetition are more dispersed and do not display a clear structuring according to diet.

Tests of homogeneity of multivariate dispersion indicated no significant differences in within-group variability among dietary treatments for either species (*T. molitor*: F = 1.21, *p* = 0.33; *G. mellonella*: F = 0.21, *p* = 0.89). This indicates that the PERMANOVA results are not driven by unequal dispersion among groups, but reflect differences in community composition.

Pairwise PERMANOVA tests controlling for experimental repetition revealed contrasting patterns between the two insect species (Table 1). In *T. molitor*, pairwise comparisons reveal a consistent dietary pattern. Control and Mixed do not differ from each other, whereas both are clearly distinct from PU and Starved. Mixed also differs from PU and Starved, while PU and Starved remain similar to each other. Together, these results indicate two main groups of diets: Control–Mixed and PU–Starved. In *G. mellonella*, none of the pairwise dietary comparisons remained significant after correction for multiple testing. Although some unadjusted *p*-values suggested differences (e.g., Control vs. PU: *p* = 0.03; Control vs. Starved: *p* = 0.04), all adjusted *p*-values were non-significant. Effect sizes (R^2^) were also moderate to low. Thus, no robust pairwise dietary differences in gut bacterial community composition were detected in *G. mellonella*.

### 3.4. Diet Impact on Gut Bacterial Community Composition and Abundance Within Insect Species

#### 3.4.1. *Tenebrio molitor*

**Figure 4:**
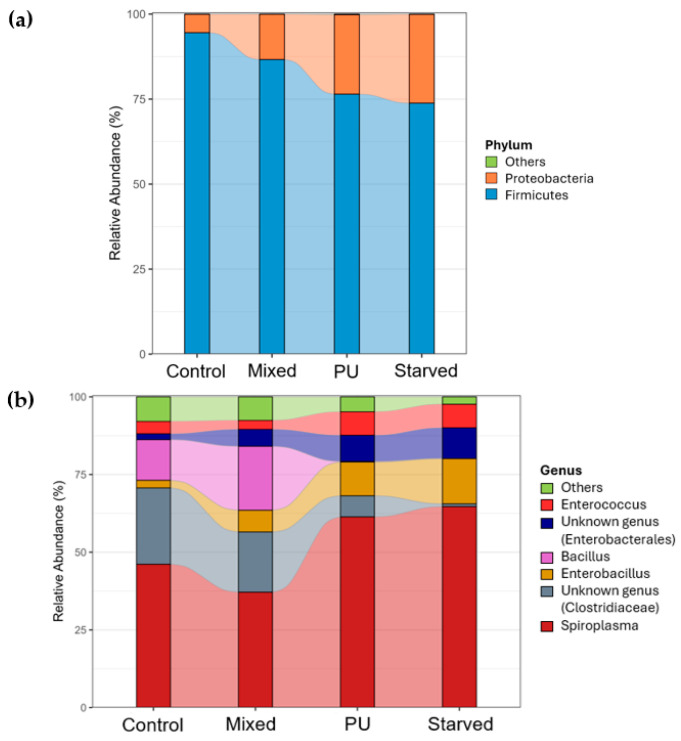
Gut bacterial community composition at phylum (**a**) and genus (**b**) levels in *T. molitor.*

At the phylum level, the gut bacterial community of *T. molitor* is dominated by Firmicutes and Proteobacteria across all dietary treatments (Figure 4a). Firmicutes represent the majority of the community in all groups, but their relative abundance decreases progressively from Control and Mixed diets to PU and Starved diets, while Proteobacteria show the opposite trend, increasing under PU and Starved conditions. At the genus level, clear shifts in community structure are observed across diets (Figure 4b). In Control and Mixed groups, the microbiota is more evenly distributed among several genera, with substantial contributions from *Enterococcus*, *Spiroplasma*, and members of Enterobacterales and Clostridiaceae families. In contrast, PU and Starved diets are characterized by a strong dominance of *Spiroplasma*, which becomes the most abundant genus and accounts for a large fraction of the total community. The relative abundance of several other genera, including *Enterococcus* and *Bacillus*, decreases under PU and Starved conditions, whereas taxa affiliated with Enterobacterales and Clostridiaceae show moderate but variable changes. Overall, Control and Mixed diets display similar taxonomic profiles, whereas PU and Starved diets share a distinct composition dominated by *Spiroplasma*, consistent with the clustering patterns observed in beta-diversity analyses.

**Figure 5:**
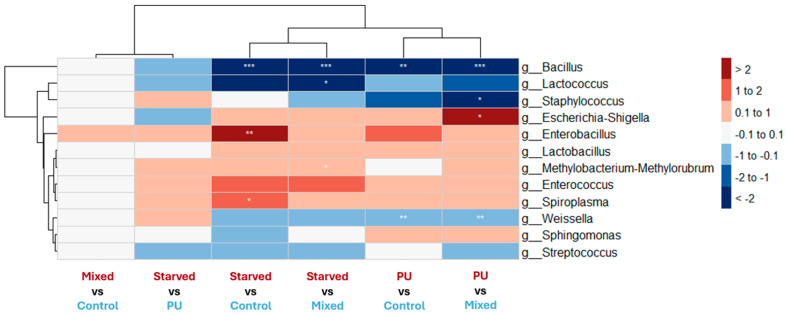
Heatmap showing DESeq2 log2 fold changes in bacterial genera in T. molitor across dietary contrasts. For each column labeled “A vs. B,” red indicates genera more abundant in A, whereas blue indicates genera more abundant in B. White indicates little or no change (|log2FC| ≤ 0.1). Only genera showing at least one significant contrast (*p* < 0.05) or |log2FC| ≥ 0.25 in at least one contrast are displayed. Stars denote statistical significance after multiple-testing correction (* *p* < 0.05, ** *p* < 0.01, *** *p* < 0.001). Hierarchical clustering is based on continuous log2FC values.

Differential abundance analysis at the genus level is consistent with both beta-diversity patterns and taxonomic composition profiles in *T. molitor* (Figure 5). Significant changes involve contrasts opposing Control and Mixed diets to PU and Starved diets, whereas no significant differences are detected between Control and Mixed, or between PU and Starved. Importantly, PU and Starved diets display parallel directional shifts in community composition compared to Control and Mixed diets. Indeed, taxa that tend to be more abundant under the PU diet also tend to be more abundant under the Starved diet when compared to Control and Mixed. For example, *Enterococcus* and *Escherichia–Shigella* show higher abundance in both PU and Starved relative to Control and Mixed, whereas genera such as *Bacillus*, *Lactococcus*, and *Streptococcus* tend to be reduced under both PU and Starved conditions. This mirrors the two main clusters observed in the PCoA and pairwise PERMANOVA analyses. Control and Mixed therefore display highly similar profiles, with only minor, non-significant differences in genus abundance.

In contrast to this general pattern, *Sphingomonas* shows a tendency toward higher abundance under the PU diet compared to Control and Mixed, while no comparable increase is observed under the Starved diet; however, this trend is not statistically significant.

#### 3.4.2. *Galleria mellonella*

**Figure 6:**
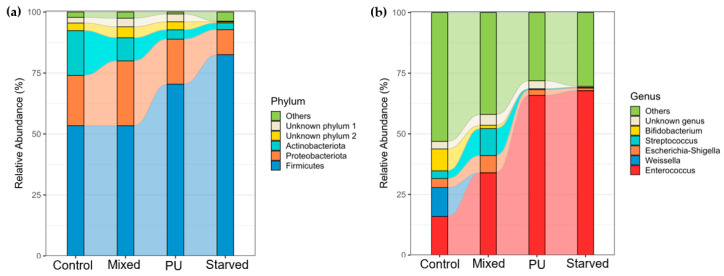
Gut bacterial community composition at phylum (**a**) and genus (**b**) levels in *G. mellonella*.

At the phylum level, the gut bacterial community of *G. mellonella* is also dominated by Firmicutes and Proteobacteria across all dietary treatments (Figure 6a). Contrary to *T. molitor*, Firmicutes increase progressively from Control and Mixed diets to PU and Starved diets, whereas Proteobacteria show a decreasing trend along the same gradient. Other phyla, including Actinobacteriota and unclassified groups, remain at relatively low and stable proportions across diets. At the genus level, marked shifts in dominant taxa are observed across dietary treatments (Figure 6b). In the Control group, the community is relatively diverse, with substantial contributions from *Weissella*, *Streptococcus*, *Escherichia–Shigella*, and *Enterococcus*. In the Mixed group, *Enterococcus* increases and becomes one of the dominant genera, while the abundance of other genera decreases. In the PU and Starved groups, this trend is even more pronounced, with *Enterococcus* strongly dominating the community and most other genera reduced to very low relative abundances.

**Figure 7:**
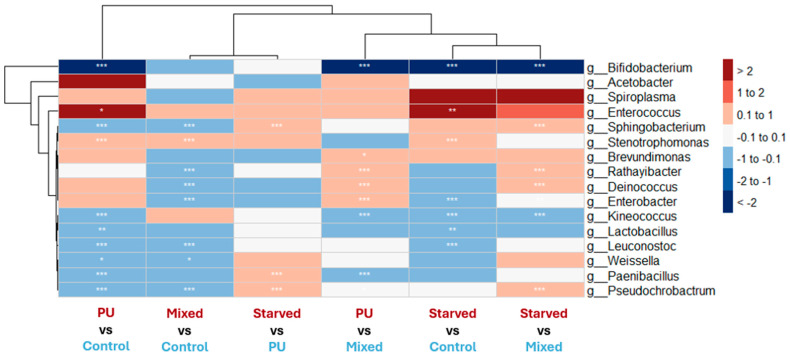
Heatmap showing DESeq2 log2 fold changes in bacterial genera in *G. mellonella* across dietary contrasts. For each column labeled “A vs. B,” red indicates genera more abundant in A, whereas blue indicates genera more abundant in B. White indicates little or no change (|log2FC| ≤ 0.1). Only genera showing at least one significant contrast (*p* < 0.05) or |log2FC| ≥ 0.75 in at least one contrast are displayed. Stars denote statistical significance after multiple-testing correction (* *p* < 0.05, ** *p* < 0.01, *** *p* < 0.001). Hierarchical clustering is based on continuous log2FC values.

In *G. mellonella*, differential abundance patterns involved a broader and more complex set of bacterial genera across dietary contrasts than those observed in *T. molitor* (Figure 7). However, several genera also displayed similar directional changes under PU and Starved conditions when compared to the Control diet.

*Enterococcus* is significantly enriched in both PU and Starved conditions relative to Control. Other genera, such as *Spiroplasma* and *Stenotrophomonas*, exhibited comparable trends toward increased abundance in PU and Starved conditions, although these changes were not consistently statistically significant. *Stenotrophomonas* was also—and the only genus—significantly enriched in Mixed diet compared to Control. Conversely, genera that tended to decrease under the PU diet also tended to be reduced in Starved larvae. Among these, *Bifidobacterium* exhibited the strongest response, while genera such as *Kineococcus* and *Lactobacillus* followed similar but weaker patterns.

Only a small number of genera show increased abundance in PU-related diets (PU and/or Mixed), without exhibiting a similar trend in Starved larvae. *Acetobacter* exhibits higher relative abundance in PU compared to Control, Mixed and Starved conditions, without being enriched in Starved. However, these differences between diets are not statistically significant. *Enterobacter* shows increased abundance in PU relative to Control and Mixed diets, reaching statistical significance only in the PU vs. Mixed comparison. At the same time, *Enterobacter* is detected at lower abundance in Mixed compared to Control, thereby weakening the evidence for a direct association between this genus and the PU diet. For both genera, these patterns are not consistently significant across all contrasts, and effect sizes vary depending on the comparison.

Other genera show more complex patterns. For example, *Rathayibacter* and *Deinococcus* are significantly enriched in PU and Starved compared to the Mixed diet, but do not show significant enrichment in PU or Starved relative to the Control diet.

### 3.5. Isolated Bacteria from T. molitor Gut

Following successive subcultures performed to obtain pure isolates, two distinct colony morphologies were observed after growth on LB agar from the 45-day PU-enriched culture. The same colony types were also recovered from the inoculated culture without PU, used as a carbon free control, whereas an additional colony type was detected exclusively in this control condition. No bacterial growth was observed in the control containing PU but no inoculum, confirming the effectiveness of the PU sterilization procedure.

**Figure 8:**
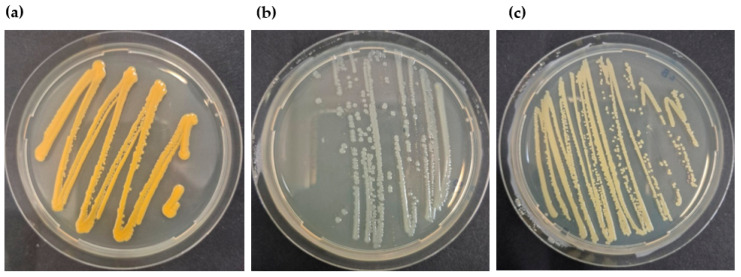
Representative colony morphologies of purified bacterial isolates grown on LB agar. (**a**) *Chryseobacterium* sp., (**b**) *Klebsiella-Enterobacter* sp., detected in both PU-enriched and inoculated control conditions, and (**c**) *Brevibacterium* sp., detected exclusively in the control without PU.

Sequencing analysis identified isolates from both PU-enriched and control cultures as belonging to the genera *Chryseobacterium* (Figure 8a) and the *Klebsiella–Enterobacter* group (Figure 8b). The additional isolate recovered from the control without PU was assigned to the genus *Brevibacterium* (Figure 8c).

## 4. Discussion

The growing interest in plastivorous insects, such as *Tenebrio molitor* and *Galleria mellonella*, is based on their ability to ingest and alter various synthetic polymers, including particularly recalcitrant plastics such as polyurethane (PU) [25,26,27]. This ability has often been interpreted as reflecting a specialized microbial activity within the gut microbiota, potentially contributing to the biodegradation of these materials [14]. However, interpretations of changes in microbial community structure or of bacteria isolated with plastic as the sole carbon source frequently rely on incomplete experimental designs in which the confounding effect of nutritional stress is insufficiently considered. By combining a plastic-only diet with a nutritionally balanced control, a mixed diet, and a starvation condition, we demonstrate that most microbial shifts observed under plastic feeding are primarily driven by severe nutritional deprivation rather than by the presence of the polymer itself.

In *T. molitor*, gut bacterial communities clearly separate into two major configurations: those associated with nutritionally adequate diets (control and mixed) and those associated with nutritionally deficient conditions (plastic-only and starvation). The strong overlap between plastic-fed and starved individuals indicates that energetic constraint is the dominant driver of microbiota restructuring, while the polymer itself exerts, at most, a secondary influence. In *G. mellonella*, dietary effects are less robust and partially masked by experimental variability, yet qualitative trends are consistent with those observed in *T. molitor*: bacterial communities associated with plastic and starvation tend to resemble each other more closely than either resembles those associated with nutritionally balanced diets. Together, these patterns indicate that, across hosts, gut microbiota primarily respond to host nutritional status, and that plastic feeding acts largely through the nutritional stress it imposes. Similar responses have been reported in other insect-microbiota systems subjected to nutrient-poor diets, where changes in bacterial composition primarily reflect microbial survival strategies rather than functional specialization [28,29].

These results directly challenge a common assumption in plastivory research, namely that enrichment of particular bacterial taxa under plastic feeding reflects their involvement in polymer degradation. In both species, many genera frequently proposed as plastic-associated or plastic-degrading candidates are also enriched under starvation [14,30,31,32]. This includes taxa such as *Enterococcus*, *Enterobacter*, *Stenotrophomonas*, and members of the *Klebsiella–Enterobacter* group, whose proliferation can be explained by tolerance to nutrient limitation, exploitation of host-derived resources, or competitive advantage in dysbiotic communities [33]. Therefore, enrichment under plastic-only diets, when compared solely to a nutritionally rich control, cannot be interpreted as evidence of functional involvement in plastic degradation. Without starvation controls, responses to famine are systematically confounded with responses to polymer exposure.

The inclusion of a mixed diet allows partial resolution of this ambiguity. A small number of taxa show responses associated with the presence of plastic even in a nutritionally adequate context, suggesting that the polymer can act as a selective factor in some cases. However, in our case, these effects are weak, inconsistent, and minor relative to the dominant effect of nutritional stress. Comparisons between plastic-fed and starved insects, which isolate polymer presence under equally severe energetic constraints, identify only a very limited set of taxa potentially responding specifically to plastic. However, taken together, these profiles indicate that while nutritional stress explains a large proportion of the changes observed under exclusive plastic feeding, the combined analysis of control, mixed, and starved diets nonetheless allows the identification of a limited number of taxa for which the presence of PU itself may contribute to microbial selection. These taxa can therefore be considered as more robust candidates for potential involvement in plastic-associated processes than those identified in the absence of such dietary controls.

These observations call for a re-evaluation of the interpretation of previous studies that identified microorganisms potentially involved in plastic degradation solely based on their enrichment under exclusive plastic diets compared to a control, nutritive diet. Several studies have reported increased abundances of genera such as *Klebsiella*, *Enterobacter* or *Pseudomonas* in *T. molitor* fed exclusively on different plastics, including polystyrene, polyurethane or polypropylene, and have consequently proposed these taxa as potential contributors to polymer degradation [25,31,32]. However, in most of these studies, the potential effect of nutritional stress associated with plastic-only diets is not explicitly addressed, even though these same taxa are known to proliferate under dysbiotic and nutrient-deficient conditions independently of exposure to xenobiotics [33]. Importantly, Yang et al. [34] recognized this limitation in their own study and explicitly pointed out that the lack of a starvation control constrained their interpretation of microbiota changes, recommending that future experiments include such controls to resolve this ambiguity. Consistent with this interpretation, another study experimentally demonstrated in an insect model (*Hermetia illucens*) that nutrient deprivation induces strong yet selective restructuring of the gut microbiota [29]. Notably, they reported the enrichment of *Sphingobacterium* and *Enterococcus* in starved larvae compared to controls, a pattern that closely mirrors our observations in *G. mellonella*, where both genera were enriched under starvation and exclusive PU feeding relative to the control diet. This variability in microbial responses across hosts and conditions highlights the necessity of a case-by-case integration of starvation controls when interpreting microbiota shifts under plastic-based diets.

Similarly, the isolation of bacteria using carbon-free media with plastic as the sole carbon source represents a valuable complementary approach to gut microbiota analyses, but it is subject to similar interpretative limitations when appropriate controls are lacking. In the absence of parallel control cultures without plastic, apparent growth or persistence may reflect survival strategies under nutrient deprivation, including the use of internal reserves or trace organic contaminants [35]. In our study, bacteria affiliated with the *Klebsiella–Enterobacter* group and the genus *Chryseobacterium* were recovered both from PU-amended cultures and from control cultures lacking plastic and any added carbon source. The recovery of an additional genus (*Brevibacterium*) exclusively in the carbon-free control further illustrates that enrichment outcomes differed between inoculated conditions and that bacterial persistence under minimal media does not necessarily depend on the presence of the polymer substrate.

These patterns mirror our microbiota results, in which several genera frequently proposed as candidates for plastic degradation were also enriched under starvation. Importantly, the recovery of these isolates under both PU-amended and carbon-free conditions indicates that their persistence alone cannot be interpreted as evidence of PU degradation. Functional validation using complementary approaches, such as monitoring polymer weight loss, spectroscopic characterization, or detection of degradation products, will therefore be required to determine whether these isolates actively contribute to polymer degradation or primarily reflect survival under extreme nutritional stress.

In the 16S rRNA sequencing dataset of *T. molitor*, members of the genera *Chryseobacterium* and *Enterobacter* were detected, although they did not show marked changes in relative abundance across dietary treatments, whereas *Brevibacterium* was not detected at the genus level. This apparent discrepancy between isolation and metabarcoding results likely reflects differences in experimental duration, as isolation was performed on a colony supplemented with PU over multiple generations, whereas microbiota profiling was based on short-term feeding treatments.

Notably, taxa previously reported as plastic-associated based on comparisons limited to plastic-fed and control-fed insects were, in our data, primarily associated with starvation rather than with plastic exposure per se, as illustrated by *Brevibacterium* and members of the *Klebsiella–Enterobacter* group [34,36], as well as *Stenotrophomonas* [37].

Taken together, these findings indicate that isolation-based approaches and microbiota analyses are conceptually complementary but methodologically constrained in similar ways, and that the integration of rigorous controls is essential to improve the robustness of candidate identification in studies of plastic-microbe interactions.

## 5. Conclusions

Our results demonstrate that microbial enrichment patterns observed under plastic-only diets cannot be interpreted independently of nutritional context. By explicitly comparing plastic feeding and starvation across two insect models, we show that several bacterial taxa frequently proposed as candidates for plastic degradation primarily respond to severe nutrient limitation rather than to the presence of the polymer itself. These responses are host- and condition-dependent, underscoring that microbial enrichment does not constitute sufficient evidence for functional involvement in polymer alteration. Together, our findings highlight the necessity of systematically integrating starvation and nutritional controls into experimental designs, and of evaluating candidate plastic-associated microorganisms on a case-by-case basis using complementary functional approaches.

## Figures and Tables

**Table 1:** Pairwise PERMANOVA comparisons of gut bacterial community composition between dietary treatments in *T. molitor* and *G. mellonella*. For each species, comparisons were performed between all pairs of diets while controlling for experimental repetition. The table reports the F statistic, proportion of variance explained (R^2^), raw and adjusted *p*-values (Benjamini–Hochberg correction), and the corresponding level of significance (ns: non-significant, ** *p* < 0.01).

Species	Comparison	F-model Statistical Value	R^2^	*p*-Value	Adjusted *p*-Value	Interpretation
*T. molitor*	Control vs. Mixed	1.02	0.08	0.372	0.372	ns
	Control vs. PU	6.85	0.41	0.003	0.005	**
	Control vs. Starved	15.80	0.61	0.002	0.003	**
	Mixed vs. PU	6.72	0.40	0.001	0.002	**
	Mixed vs. Starved	16.72	0.62	0.001	0.002	**
	PU vs. Starved	1.83	0.16	0.09	0.112	ns
*G. mellonella*	Control vs. Mixed	1.42	0.15	0.16	0.24	ns
	Control vs. PU	2.85	0.25	0.03	0.12	ns
	Control vs. Starved	3.44	0.25	0.04	0.12	ns
	Mixed vs. PU	1.02	0.10	0.35	0.35	ns
	Mixed vs. Starved	1.62	0.12	0.13	0.24	ns
	PU vs. Starved	1.09	0.06	0.28	0.34	ns

## Data Availability

Raw sequencing data have been deposited in the NCBI Sequence Read Archive under BioProject PRJNA1406142.

## Supplementary Materials

The following supporting information can be downloaded at: https://www.mdpi.com/article/10.3390/microorganisms14030649/s1. Figure S1: Fourier-transform infrared (FTIR) spectrum of the polyurethane (PU) material used in this study; Figure S2: Raman spectra of particles recovered from frass of *Tenebrio molitor* and *Galleria mellonella* larvae fed with PU, compared with reference PU; Figure S3: Optical microscopy images of particles recovered from frass of PU-fed larvae compared with pristine PU material.

## Abbreviations

The following abbreviations are used in this manuscript:

PU	Polyurethane
PBS	Phosphate-Buffered Saline
MSM	Minimal Salt Medium
