# Supplementary material for: Assessing the Impact of Nutritional Stress on the Identification of Plastic-Associated Bacteria in Insect Gut Microbiota
Source: Microorganisms. 2026 Mar 13;14(3):649. doi: 10.3390/microorganisms14030649 (PMC13029416; doi:10.3390/microorganisms14030649)
Supplement: Supplementary file 1 [file microorganisms-14-00649-s001.zip › Figure S1.pdf]

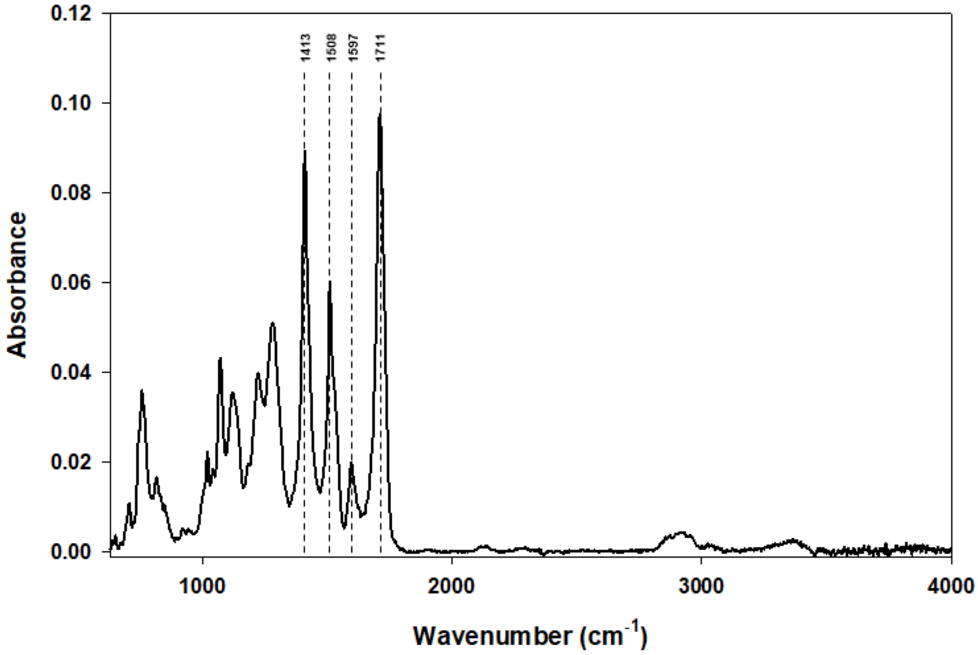

Figure S1. Fourier-transform infrared (FTIR) spectrum of the commercial polyurethane (PU) foam used in this study, obtained on a Ge crystal. Major absorption bands characteristic of polyurethane functional groups are highlighted with dashed lines. Characteristic urethane bands are observed at 1711 cm^{-1} (C=O stretching) and 1508 cm^{-1} (N–H in-plane bending). An aromatic C=C stretching band appears at 1597 cm^{-1}. The band at 1413 cm^{-1} corresponds to C–N stretching of isocyanurate rings, typical of PIR materials. The spectrum was used to confirm the polymer family of the material prior to insect exposure.
