# Supplementary material for: Assessing the Impact of Nutritional Stress on the Identification of Plastic-Associated Bacteria in Insect Gut Microbiota
Source: Microorganisms. 2026 Mar 13;14(3):649. doi: 10.3390/microorganisms14030649 (PMC13029416; doi:10.3390/microorganisms14030649)
Supplement: Supplementary file 1 [file microorganisms-14-00649-s001.zip › Figure S2.pdf]

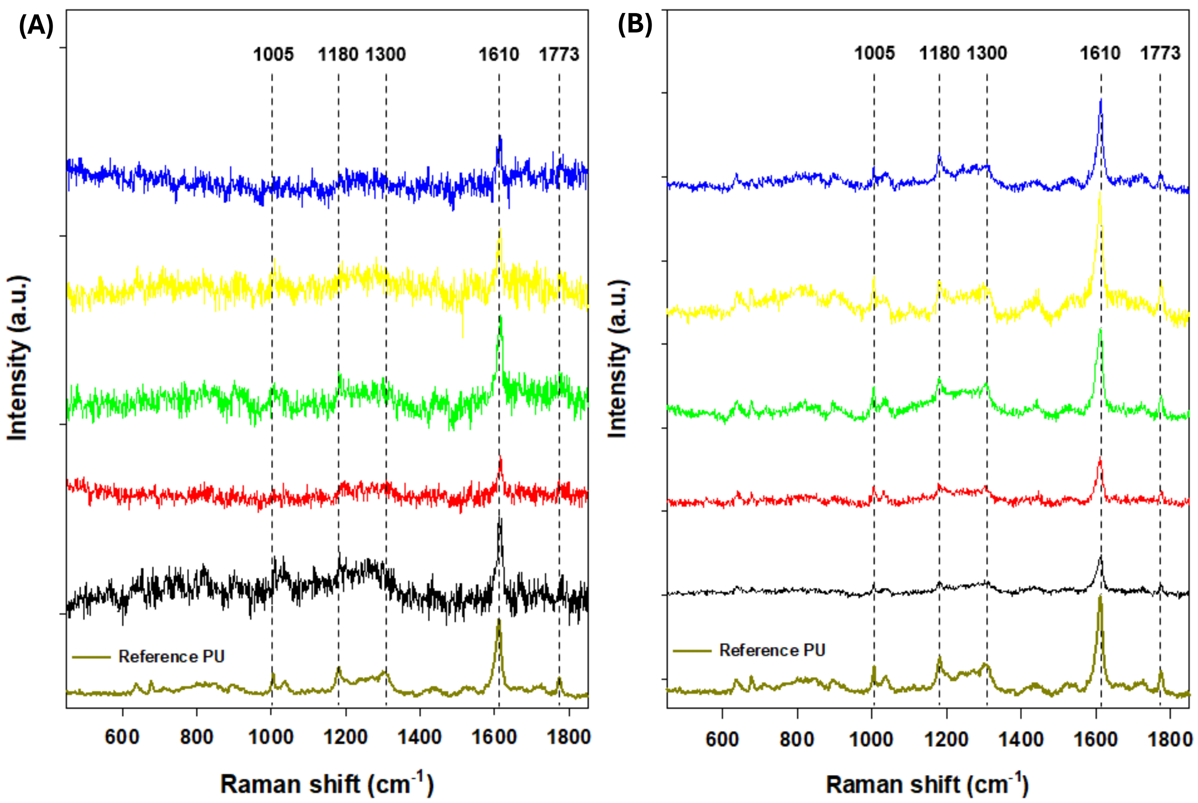

Figure S2. Representative Raman spectra obtained from particles recovered from frass of PU-fed larvae of *Tenebrio molitor* (A) and *Galleria mellonella* (B) after digestion and filtration, compared with the reference PU spectrum. For each specie, spectra were recorded on five independent particles (shown in different colors) originating from different individuals and experimental replicates. The bottom spectrum in each panel corresponds to the reference PU material. Matching spectral features confirm the polymeric identity of the recovered particles.
