# Supplementary material for: Assessing the Impact of Nutritional Stress on the Identification of Plastic-Associated Bacteria in Insect Gut Microbiota
Source: Microorganisms. 2026 Mar 13;14(3):649. doi: 10.3390/microorganisms14030649 (PMC13029416; doi:10.3390/microorganisms14030649)
Supplement: Supplementary file 1 [file microorganisms-14-00649-s001.zip › Figure S3.pdf]

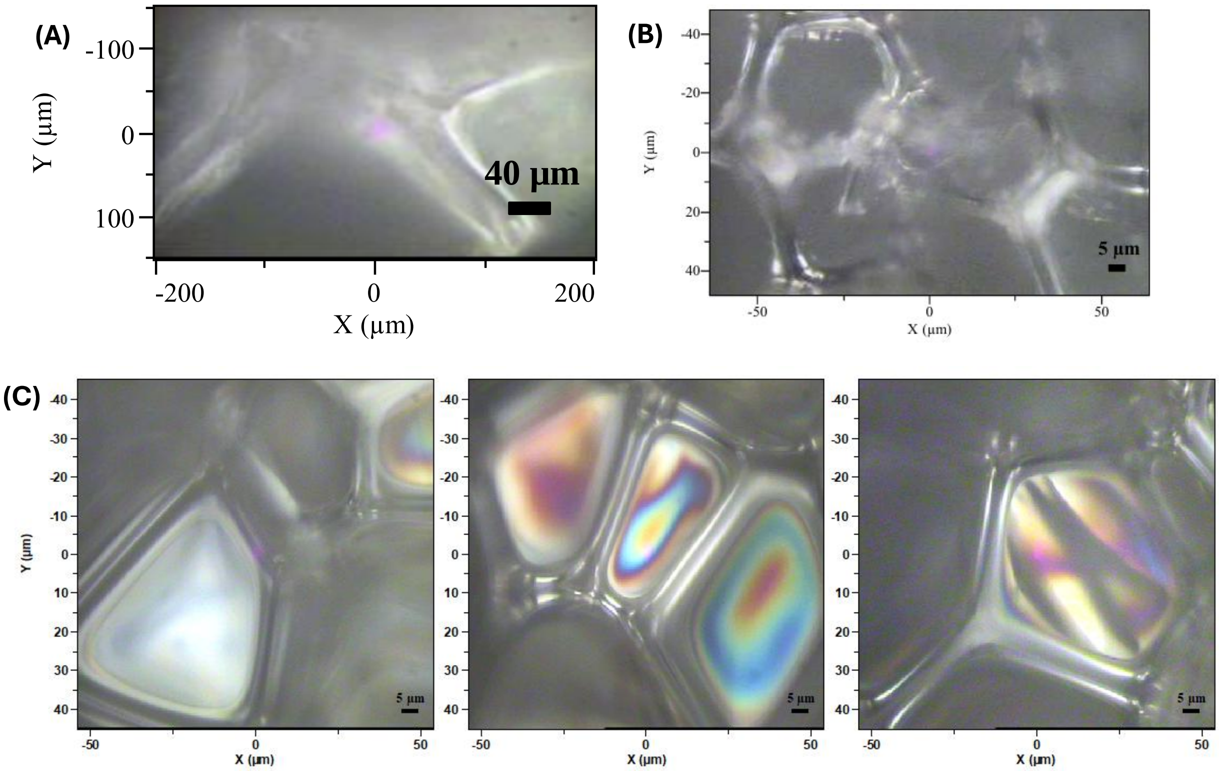

Figure S3. Optical microscopy images of particles recovered from frass of *Tenebrio molitor* (A) and *Galleria mellonella* (B) larvae exposed to a PU-only diet after chemical digestion and filtration, compared with the pristine polyurethane (PU) material (C). The alveolar structure is characteristic of the PU foam.
